# Role of Serum Vitamin D, Interleukin 13, and microRNA-135a in Hepatocellular Carcinoma and Treatment Failure in Egyptian HCV-Infected Patients Receiving Direct Antiviral Agents

**DOI:** 10.3390/v13102008

**Published:** 2021-10-06

**Authors:** Mohamed E. Ali, Hamada M. Halby, Mamdouh Yones Ali, Elham Ahmed Hassan, Mohamed A. El-Mokhtar, Ibrahim M. Sayed, Marwa M. Thabet, Magdy Fouad, Ahmed M. El-Ashmawy, Zainab Gaber Mahran

**Affiliations:** 1 Department of Microbiology and Immunology, Faculty of Pharmacy, Al-Azhar University, Assiut 71524, Egypt; elkady4work@azhar.edu.eg (M.E.A.); hamadahalby@ymail.com (H.M.H.); mamdouhyones75@yahoo.com (M.Y.A.); 2 Department of Gastroenterology and Tropical Medicine, Faculty of Medicine, Assiut University, Assiut 71515, Egypt; elham.abdelhalem@med.aun.edu.eg; 3 Department of Medical Microbiology and Immunology, Faculty of Medicine, Assiut University, Assiut 71515, Egypt; elmokhtarma@aun.edu.eg (M.A.E.-M.); Ibrahim.ibrahim@aun.edu.eg (I.M.S.); 4 Department of Clinical Pathology, Faculty of Medicine, Assiut University, Assiut 71515, Egypt; d_marwa25@yahoo.com; 5 Hepato-Gastroenterology Unit, Tropical Medicine Department, Faculty of Medicine, El-Minia University, Minya 61519, Egypt; Drmagdyf@yahoo.com; 6 Gastroenterology and Hepatology Unit, Department of Internal Medicine, Faculty of Medicine, Assiut University, Assiut 71515, Egypt; dr.ashmawy82@gmail.com

**Keywords:** HCV, prognostic marker, IL-13, vit D, miRNA-135a, HCC

## Abstract

Direct-acting antivirals (DAAs) are used for hepatitis C virus (HCV) treatment. However, treatment failure and hepatocellular carcinoma (HCC) development following treatment was reported. In this study, we assessed the role of serum vitamin D, interleukin 13 (IL-13), and microRNA-135a in the prediction of treatment failure with DAA and HCC development among Egyptian HCV-infected patients. A total of 950 patients with HCV-related chronic liver disease underwent DAA treatment. Before DAAs, serum vitamin D and IL-13 were determined by ELISA, and gene expression of miRNA-135a was assessed in serum by real-time PCR. The predictive abilities of these markers were determined using the receiver operating characteristic (ROC) curve. Sustained virological response (SVR) was achieved in 92.6% of HCV-infected patients (responders). High viral load, IL-13, miRNA-135a, and low vitamin D levels were associated with treatment failure and HCC development. HCC development was recorded in non-responders, but not in the responders (35.7% vs. 0% *p* < 0.001). In conclusion: serum IL-13, Vitamin D, and miRNA-135a could be potential biomarkers in monitoring DAA treatment and HCC prediction. DAAs-induced SVR may decrease the incidence of HCC.

## 1. Introduction

Chronic hepatitis C (CHC) infection is a global disease that has a variable course. It is a main cause of liver cancer and cirrhosis [1,2]. However, there is no available vaccine against HCV [3]. The initial recommended treatment depends on the HCV genotypes, treatment status, and the severity of liver disease [4,5]. Direct antiviral agents (DAAs) have achieved sustained virological response (SVR) and recovery in more than 90% of HCV-infected patients [6,7]. HCV resistance-associated substitutions (RASs) are selected and could persist for years after treatment failure, and may adversely impact retreatment responses. Newly approved regimens with improved potency and resistance profiles are less impacted by resistance and provide the best retreatment options for patients who previously failed DAA therapy [8,9]. Knowledge of factors, such as the presence of cirrhosis and prior treatment regimens, remain key to optimize retreatment approaches [9]. The combination of sofosbuvir, velpatasvir, and voxilaprevir may be used in those patients who were not cured by sofosbuvir or other drugs that inhibit NS5A [10]. However, resistance of HCV to DAAs can develop due to the loss of re-reading activity of RNA polymerase and rapid replication rate of the virus, which leads to the development of genetically distinct viral variants named “quasispecies” [11,12]. Dominant variants can be found within the viral quasispecies along with less-fit variants that are present at lower frequencies. Previous studies showed that the impact of antiviral therapy could be affected by the presence of minor resistance-associated variant populations (RAVs) at the start of treatment [13]. Such variants can become dominant because of selective pressure exerted by antiviral drugs, resulting in virological breakthroughs during treatment or relapse after cessation of treatment [14,15].

Vitamin D (vit D) has anti-inflammatory and anti-fibrotic functions that can affect the treatment outcome and minimize HCV-mediated liver disease [11,16,17]. Previous studies showed that vitamin D and its metabolites can synergize interferon treatment and inhibit HCV replication in vitro [18,19]. Vitamin D has a positive role in the virological response to therapy, and its level was positively correlated with achieving SVR [20,21,22]. A link between vit D and liver disease is reported. Therefore, liver diseases including CHC may be responsible for low serum levels of vit D. Consequently, the deficiency in vit D could lead to poor response to peg interferon (PEG-INF α) and ribavirin (RBV) in HCV genotype 1 infection [23].

Interleukin-13 (IL-13) is a glycosylated polypeptide of the family of IL-13/IL-4 [24,25]. IL-13 has an anti-inflammatory effect on cytokines and chemokines, and it suppresses macrophage activity [26,27]. Its proteins and antibodies are important mediators of immunoregulatory processes in various cell types [27,28,29]. Elevated expression of IL-13 was recorded in the liver tissues of chronic HCV patients. Furthermore, Weng et al. revealed significantly enhanced serum levels of IL-13 in chronic HCV-infected patients compared with healthy controls, and IL-13 protein is linked with the fibrotic stage in HCV-infected patients and steatosis/steatohepatitis [30]. Moreover, the crucial roles of IL-13 have been studied in tumors as it was reported that IL-13 was over expressed in tumor tissues and targeting IL-13 in cancer may have a potent role in cancer immunotherapy [31,32]. In addition, previous studies showed that IL-13 levels were significantly higher prior to DAA therapy in the patients who later developed HCC and stayed higher at each subsequent time point [33,34,35].

miRNA-135a is elevated broadly across many cancer types and a candidate driver of HCV-associated HCC [36]. miRNA-135a modulates HCV by affecting the viral core protein expression and replication [36]. However, the role of miRNA-135a in HCV infection HCV-associated complications has not been studied yet. 

HCC is a global health dilemma. Primary liver cancer is the seventh most-prevalent cancer that can lead to a high mortality rate [37,38]. The annual incidence of HCC in cirrhotic patients is 3–7% [2,39]. The high rate of SVR with DAA treatment may raise expectations for significant reduction in this incidence, as previously observed with IFN-based regimens. However, the impact of DAA-based regimens on the occurrence of HCC in cirrhotic patients after curative treatment is controversial. Several studies reported unexpectedly high occurrence or recurrence rates of HCC after DAA treatment [40,41,42]. Nault and Colombo reported that HCC occurs annually in a rate of 0.4–2% in advanced liver disease following the occurrence of SVR [43]. Recent reports of the increased incidence of recurrent and de novo HCC after DAA use raised great concern. Following the publication of these reports, several other studies have confirmed the increased occurrence of HCC after DAA use [40,41,44,45]. Several predictive factors associated with HCC development after DAA treatment were previously determined such as angiopoietin-2 (ANGPT2), MIG, IL22, TRAIL, APRIL, VEGF, IL3, TWEAK, SCF, and IL21 [35,46]

In this study, we evaluated the association between the selected biomarkers: Vit D, IL-13, and miRNA-135a, and treatment outcome of HCV with DAAs and the risk of development of HCC in Egyptian HCV-infected patients who received DAA therapy in the form of “sofosbuvir and daclatasvir ± ribavirin”. The previous regimen is an acceptable protocol provided by the Egyptian Ministry of Health through the National Committee for Control of Viral Hepatitis (NCCVH) and showed efficacy over 90% [47].

## 2. Materials and Methods

### 2.1. Study Design

This study was carried out at the Medical Research center, Faculty of Medicine, Assiut University, Egypt from December 2018 to November 2020. This work has been approved by the Local Ethical Committee of the Faculty of Medicine, Assiut University (IRB no. 17300237 and 17300672). All participants were adults and all of them provided written informed consent before collection of samples.

### 2.2. Study Subjects

A total of 950 patients with treatment-naïve HCV-related chronic liver disease in addition to 50 healthy controls were enrolled in the study. Patients were selected from those attending the AL-Rajhi Liver Center, Assiut University Hospital, Egypt, to dispense DAA treatment during the 100 million healthy lives campaign. AL-Rajhi Liver Center is a large referral tertiary center that provides surveillance, counselling, investigation, and treatment for HCV-infected patients in governorates of Upper Egypt, and it is one of the hospitals that participated in the campaign. Eligible patients were diagnosed with CHC infection based on the presence of anti-HCV antibody levels and detectable serum HCV RNA. The enrolled patients were either non-cirrhotic or cirrhotic. Cirrhosis was determined by fibrosis-4 (FIB-4) score with a FIB-4 >3.25 for advanced fibrosis/cirrhosis and transient elastography (TE, FibroScan, Echosens, Paris, France) with a liver stiffness ≥14.6 kPa [48].

The healthy controls (*n =* 50) were negative to HBV and HCV markers, and they had no hepatic diseases and were sex- and age-matched with patients. They were selected randomly from outpatient clinic and relatives of the patients. We excluded patients treated with interferon-based regimens or immunomodulating agents. Additionally, patients who were co-infected with hepatitis A and B viruses or human immunodeficiency viruses, those having alcohol or drug-induced liver diseases, those with positive anti-schistosomal antibodies, and those with evidence of HCC before DAA therapy were excluded from this study. 

Before starting the DAA therapy, a thorough medical history and clinical examination were obtained, and all patients were screened using abdominal ultrasound. Blood samples were collected for measuring serum vit D, IL-13, and miRNA-135a levels. In addition, HCV load was determined. Patients received DAA therapy in the form of “sofosbuvir (400 mg) and daclatasvir (60 mg) ± ribavirin (1000–1200 mg according to the body weight” for 12 weeks based on the protocol provided by the National Committee for Control of Viral Hepatitis in Egypt (NCCVH guidelines for the management of adult patients with HCV infection) available at the time of enrolment [47]. The combination of sofosbuvir and daclatasvir remains an acceptable option, according to the EASL Recommendations for Treatment of Hepatitis C 2016 [49,50,51,52,53]. SVR was defined as non-detectable HCV-RNA in serum after six months of treatment. Previous studies reported that SVR at 12 weeks post-treatment (SVR12) is as valid as 24 weeks post-treatment (SVR24) as an efficacy endpoint due to its high rate of concordance with SVR24 [54,55]. However, a recent study performed on Egyptian HCV-infected patients showed that SVR24 is a reliable initial endpoint of sofosbuvir-based treatment response monitoring, especially in cases of predication of the relapsing [55]. Following DAA therapy, they were followed up for monitoring treatment response (six months after the end of treatment) and screening for HCC (within one year after the end of treatment). Patients were divided into two groups: patients with SVR (responders) and patients who were non-SVR (non-responders). Diagnosis of HCC was based on elevated alpha fetoprotein-L3 (AFP-L3), and triphasic computed tomography scan according to EASL Clinical Practice Guidelines [56].

### 2.3. Sample Processing

Blood samples were collected into plain tubes labeled with the patient’s name, sex, age, and the date of collection. Serum was isolated from blood and divided into two tubes; the first tube was used for determination of serum level of vit D and IL-13 and the second tube was used for RNA extraction to be used in quantitative real-time PCR reactions (RT-qPCR) for estimating the level of miRNA-135a gene expression.

### 2.4. Quantitative Real-Time Polymerase Chain Reaction (RT-qPCR) 

Total RNA was isolated from serum samples using miRNA isolation Kit (Applied Biosystems, Foster City, CA, USA) following the manufacture’s instruction. cDNA was synthesized using Universal cDNA Synthesis Kit (Exiqon, Cat no. 203300). 10 µL of reverse transcription reactions was used for Syber green based miRCURY LNA Universal RT microRNA PCR assay. U6 was used as an internal reference. The primers for miR-135a and U6 were as follows: miR-135a (Forward: 5′-ACACTCCAGCTGGGTATGGCTTTTTATTCCT-3′; Reverse: 5′-GGTGTCGTGGAGTCGGCAA-3′); U6 (Forward: 5′-CTCGCTTCGGCAGCACA-3′; Reverse: 5′-AACGCTTCACGAATTTGCGT-3′). PCR conditions for miR-135a amplification were 95 °C for 20 s, followed by 40 cycles of 95 °C for 10 s and 60 °C for 20 s, with a final incubation at 70 °C for 5 s. qRT-PCR was carried out under optimal conditions. Expression level of miR-135a was normalized to U6 and determined using the 2-DDCt method [57].

### 2.5. Determination of Vit D by ELISA

Vitamin D was determined in serum using Human Vitamin D Promokine (direct) ELISA kit (PromoCell GmbH, Sickingenstr. Heidelberg, Germany) according to the manufacturer’s instructions. Briefly, 20 µL of standards/controls/serum was added into the wells. Strips were incubated for 45 min at room temperature on horizontal shaker, then 150 µL of anti-vitamin D antibody (AB) was added into each well. The plate was covered and incubated for 45 min at room temperature on a horizontal shaker. After that, the content of each well was discarded, and plate was washed 5 times using 250 µL of wash buffer. After the final washing step, 200 μL of conjugated CONJ was added into each well, and the plate was incubated for 45 min at room temperature on a horizontal shaker. The washing steps were repeated again and then 200 µL of substrate was added into each well and incubated for 10–15 min at room temperature in the dark. The reaction was stopped by addition of 50 µL stop solution into each well. Absorption was determined immediately with an ELISA reader at λ 450 nm. 

### 2.6. Determination of Human IL-13 by ELISA 

IL-13 was determined in serum using RayBio^®^ Human IL-13 ELISA Kit (RayBiotech, Peachtree Corners, GA, USA) according to the manufacturer’s instructions Briefly, 100 µL of each standard and sample was added into appropriate wells, then the wells were incubated for 2.5 h at room temperature with gentle shaking. After the washing step. A total of 100 µL of 1X prepared biotinylated antibody was added to each well and incubated for 1 h at room temperature with gentle shaking. Then, the plate was washed and 100 µL of prepared Streptavidin solution was added to each well and incubated for 45 min at room temperature with gentle shaking. After the washing step, 100 µL of TMB one-step substrate reagent was added to each well and incubated for 30 min at room temperature in the dark with gentle shaking. After the color development, 50 µL of stop solution was added to each well, and the plate was read using ELISA reader at λ 450 nm.

### 2.7. Quantification of HCV Viral Load

HCV-RNA was determined in serum using artus^®^ HCV RG RT-PCR Kit (QIAGEN GmbH, QIAGEN Strasse, Hilden, Germany) according to the manufacturer’s instructions. Briefly, desired number of PCR tubes were placed into the adapters of the cooling block. The reaction mix typically contains all the components needed for PCR except the sample. 30 µL of the master mix were added into each PCR tube, then 20 µL of the eluted sample RNA were added. Correspondingly, 20 µL of at least one of the quantitation standards (Hep. C Virus RG QS 1–4) must be used as a positive control and 20 µL of water (water, PCR grade) as a negative control. The PCR tubes were closed. The locking ring (accessory of the Rotor-Gene Instrument) was placed on top of the rotor to prevent accidental opening of the tubes during the run. For the detection of HCV RNA, a temperature profile was created according to manufacturer’s instructions.

### 2.8. Statistical Analysis

All statistical analyses were done by using GraphPad Prism 8.4.0, Statistical Package for the Social Sciences (SPSS) for Windows version 16 (SPSS Inc., Chicago, IL, USA) and MedCalc program. The Kolmogorov–Smirnov test of normality was used to test the normality of data. The quantitative data were expressed as mean ± standard deviation (SD) or median and the range (minimum–maximum) for normally or abnormally distributed data, respectively. They were compared using Student’s *t*-test, ANOVA or Mann–Whitney U-test or Kruskal–Wallis test for two or more groups of normally or abnormally distributed data, respectively. Qualitative data were expressed as a percentage and compared using chi-squared (χ*2*) or Fisher’s exact probability test. The receiver operating characteristic curves (ROC) were plotted to measure the performance of investigating parameters in predicting the treatment failure and HCC and to select its optimal cut-off value at which the sensitivity, specificity, and positive likelihood ratio. All tests were two-tailed and *p*-values < 0.05 were considered significant.

## 3. Results

### 3.1. Characteristics of the Study Population According to the Treatment Response

A total of 950 patients with HCV-related chronic liver disease (451 males and 499 females) were included in this analysis. The patients received DAA therapy (sofosbuvir and daclatasvir ± ribavirin” for 12 weeks), and they were followed up 6 months after the end of therapy to monitor the therapy outcome. Out of 950 patients, 880 patients (92.6%) achieved SVR (responders), and HCV RNA was not detectable in the serum of these patients, while 70 patients (7.36%) did not achieve the SVR (non-responders). We assessed the demographic and clinical characteristics of responders and non-responders to identify the risk factors associated with the therapy failure. There was no significant difference between responders and non-responders in terms of age, sex, and smoking (Table 1), while we found that comorbidities such as diabetes and hypertension were associated with the treatment failure with DAAs. A total of 62.9% of the non-responders were cirrhotic at the start of the therapy compared with 15% of the responders who were cirrhotic patients (Table 1). The serum level of IL-13 was higher in HCV patients compared with the healthy controls with a significantly higher level in non-responders compared with responders. Moreover, elevated expression of miRNA-135a (mean fold change was 4.6 and 2.1 in non-responders and responders, respectively, compared with healthy controls) led to unresponsiveness to treatment (Table 1). In contrast, the serum level of vit D was lower in chronic HCV-infected patients compared with healthy controls, with a significantly reduced level in non-responders compared with the responders. Responder patients had a significantly lower HCV load (4.7 × 10^5^ IU/mL) at the start of therapy compared with non-responders (9.6 × 10^5^ IU/mL). None of the responders had developed HCC during the follow-up period, while 35.7% (25/70) of the non-responders had developed HCC.

**Figure 1:**
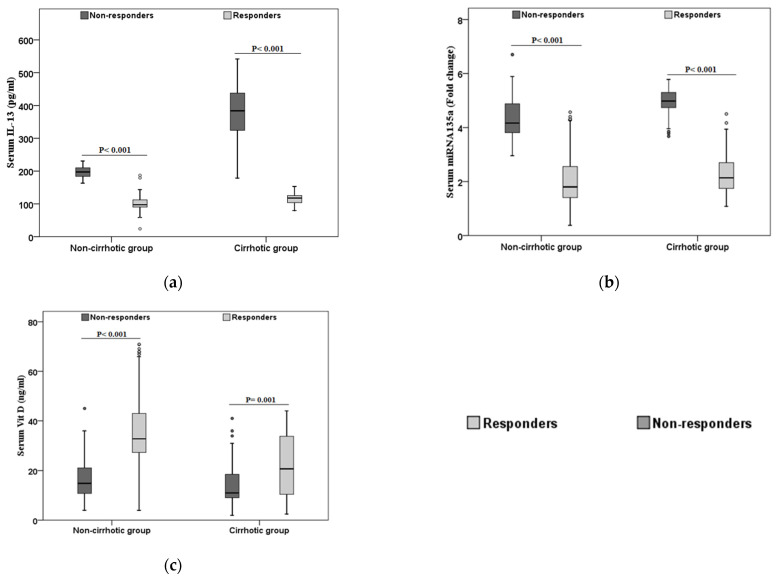
Comparison between laboratory parameters in different patients’ groups. Comparing the laboratory parameters between the cirrhotic and non-cirrhotic patients according to the therapy response (**a**): IL-13. (**b**): miRNA-135a. (**c**): Vit D. Responders are represented as light grey and non-responders are represented by dark grey.

Then, we assessed the levels of these parameters (IL-13, vit D, and miRNA-135a) in responders and non-responders in both cirrhotic and non-cirrhotic patients (Figure 1). In cirrhotic patients, non-responders had a significantly elevated serum level of IL-13, and miRNA135a compared with responders (Figure 1a,b), while the serum level of vit D was lower in non-responders LC patients compared with responder LC patients (Figure 1c). Similarly, in non-cirrhotic patients, non-responders had higher serum levels of IL-13, and miRNA-135a and lower serum level of vit D compared with responders (Figure 1).

### 3.2. Characteristics of the Non-Responders According to the HCC Development

Out of 70 non-responders, 25 patients (35.8%) had developed HCC following DAA therapy, while 45 patients (64.2%) had not developed HCC. We assessed the demographic, and clinical characteristics of non-responders to identify the risk factors and predictive markers for HCC development following the therapy failure. We found that age, sex, smoking, and comorbidities such as diabetes and hypertension were not significantly different between non-responders who developed HCC and non-responders without HCC (Table 2), while the serum level of IL-13 and miRNA-135a were significantly higher at the start of therapy in the non-responders who developed HCC after that compared with non-responders who did not develop HCC (Table 2). Additionally, the HCV viral load was higher in HCC patients than patients without HCC (Table 2).

### 3.3. Diagnostic Accuracy of IL-13, miRNA-135a, and Vit D to Predict the Treatment Failure and HCC Development

**Figure 2:**
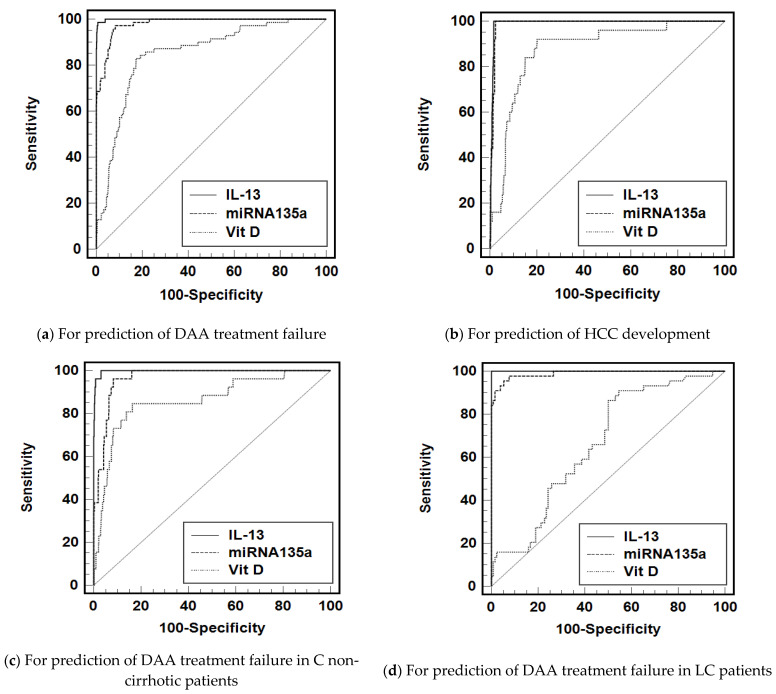

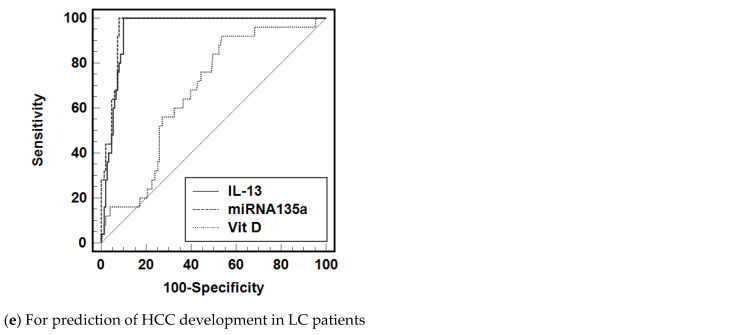
Diagnostic accuracy of IL-13, miRNA-135a and vit D to predict treatment failure and HCC with the best predictive cut offs. (**a**) ROC analysis to discriminate between responders and non-responders using IL-13, miRNA-135a, and vit D. (**b**) ROC analysis to predict the development of HCC among HCV-infected patients using IL-13, miRNA-135a, and vit D. ROC curves to predict DAA therapy failure in non cirrhotic (**c**) and cirrhotic patients (**d**) using the previous mentioned markers. (**e**) ROC curve to predict the development of HCC among cirrhotic patients.

We assessed the diagnostic utility of IL-13, miRNA-135a, and vit D to predict the therapy failure and the development of HCC. To this end, we generated the receiver operating characteristic curve (ROC) that plots sensitivity versus 1-specificity to evaluate the overall performance of each marker. Using the serum levels of IL-13 as a cutoff to discriminate between responders and non-responders, the AUC was 0.999 (95% CI (0.998–1)) and serum IL-13 > 135.9 pg/mL had a sensitivity of 100% and specificity of 96% to differentiate between responders and non-responders (Table 3, Figure 2a). The AUC of serum miRNA135a was 0.978 (95% CI (0.967–0.988)) and the cut-off value of serum level of miRNA135a >3.562-fold change could differentiate between responders and non-responders with 97% sensitivity, 91% specificity, and likelihood ratio of 11.7. The serum level of vit D <21.13 ng/mL has 83% sensitivity and specificity, with AUC = 0.851 (95% CI; 0.807–0.895) (Table 3, Figure 2a).

Then, we assessed the possibility of using these markers to predict the development of HCC. ROC analysis revealed that the AUC was 0.992 (95% CI; 0.987–0.997), 0.973 (95% CI; 0.964–0.989), and 0.872 (0. 809–0.935) for IL-13, miRNA135a, and vit D, respectively. A serum level of IL-13 of >249 pg/mL can differentiate between the non-responders who developed HCC and HCV patients who did not develop HCC with a 100% sensitivity and 98.4% specificity. Similarly, the cut-off value of serum miRNA135a >3.66-fold change can discriminate between HCC and non-HCC with 100% sensitivity and 89% specificity. Finally, the serum level of vit D <21.04 ng/mL can differentiate the HCC patients from non-HCC patients with an 80% specificity and 92% sensitivity (Table 3, Figure 2b). Additionally, these markers can be used to predict the therapy efficacy among cirrhotic or non-cirrhotic patients (Table 3).

## 4. Discussion

HCV infection is one of the major health problems worldwide. Egypt has the highest prevalence of HCV infection [58,59]. Previously, HCV infection was treated with PEG-INF α ± RBV [60]. DAAs are used for the treatment of this infection regardless of the HCV genotypes [61,62,63]. However, failure of HCV treatment with DAAs was recorded [64,65,66]. DAAs have made a revolution in HCV treatment with promising reduction of HCV infection and disease morbidities. However, treatment failure is reported in about 5–15% of patients treated with DAA-based combination regimens. Therefore, a quadruple regimen of sofosbuvir, daclatasvir, and simeprevir with a weight-based ribavirin in chronic HCV DAA-experienced patients were used and recommended. Retreatment of HCV genotype 4 patients with quadruple therapy is a good therapeutic option and achieves high response rates with minimal side effects [67]. In Egypt, HCV-infected patients received DAA therapy in the form of “sofosbuvir and daclatasvir ± ribavirin”, which is the common therapy protocol approved by the Egyptian Ministry of Health [50]. Several biomarkers were reported with HCV therapy failure and HCC development such as IL-6, IL-17, vit D, etc. [68,69,70,71,72]. However, the biomarkers associated with DAA failure and HCC development following DAA are not completely known.

Chronic HCV-infected patients who received DAAs were divided into two groups: responders and non-responders, according to the SVR. The non-responders were divided based on the complications associated with therapy failure into patients who developed HCC and patients without HCC. Our results revealed that diabetes, hypertension, and liver cirrhosis were risk factors for therapy failure. Moreover, the HCV load, elevated serum level of IL-13, and upregulation of miRNA-135a expression was linked to the unresponsiveness to DAA therapy and persistent infection. Similarly, several studies reported the presence of a significant link between diabetes, hypertension, and viral load with HCV therapy failure [73,74,75,76,77]. The severity of liver disease at the start of therapy such as liver cirrhosis might be one of the causes of the treatment failure with DAAs. Additionally, higher DAA treatment failure rates were observed in patients with decompensated cirrhosis and in liver transplant recipients. These predictive baseline factors should be assessed to determine the appropriate time points of DAA therapy [78]. A shift in the immune response to an anti-inflammatory status and the development of pro-fibrogenic chemokine and cytokine profiles are reported with persistent infection and impaired viral clearance. A significantly higher level of IL-4 and IL-13 was recoded in HCV patients who did not achieve SVR and/or acquired HCC [30,79,80,81,82]. IL-13 was significantly higher at initial viremia and in persistent infection, while its level is low after the viral clearance [79,83]. IL-13 promotes liver granuloma formation by acting on macrophages/neutrophils independently [84]. HCV infection upregulates miRNA-135a expression that mediates the viral replication and contributes to HCV-associated HCC [36]. Therefore, upregulation of miRNA135a expression is associated with resistant to DAA therapy.

Additionally, we showed that a lower serum level of vit D in chronic HCV-infected patients was associated with therapy failure and the development of HCC. In a parallel line, Abd Allah et al. reported that CHC is associated with vitamin D deficiency but iron overload, resulting in a reduced level of hepcidin. Treatment with sofosbuvir/daclatasvir increases hepcidin, thereby reducing iron levels and increasing vit D [85]. A study by [19] showed that vit D reduces HCV protein production in cell culture synergistically with IFN-α. Vitamin D, along with IFN-α, enhances gene expression, leading to HCV clearance in vivo. In contrast, one study reported that treatment response with DAAs does not depend on the pretreatment vitamin D levels [86]. Additionally, higher vitamin D status was not beneficially associated with responses to therapy; if anything, patients with higher vitamin D concentrations were less likely to attain SVR and do not support a role for vitamin D supplementation as an adjuvant therapy for HCV [87]. In addition, vitamin D level has no impactful role with DAA therapy in HCV-infected patients. However, among patients who achieved an SVR for HCV, some patients (48%) did not regress after SVR, and some (6%) even worsened, with an increased risk for hepatocellular carcinoma [88]. Backsted et al. [89] reported the prevalence of vitamin D deficiency was higher in hepatitis-C–related cirrhotic cohorts compared with non-cirrhotic patients and correlated with components of hepatic function. However, the pretreatment with vitamin D level nor the change of its level during the therapy did not lead to an increased rate of SVR.

In addition, we evaluated the risk factors and prognostic markers for the acquisition of HCC after DAA treatment. We found that high viral load, elevated IL-13 and miRNA135a levels, and reduced serum levels of vit D were associated with HCC development following the DAA failure. HCV infection can progress to cirrhosis-related HCC [90]. Several studies showed a correlation between miRNA-135a levels and malignant behaviors in HCC patients, which made it a useful target for prognostic and therapeutic uses [91,92]. In addition, miRNA-135a plays a role in promoting portal vein tumor thrombus (PVTT) by promoting metastasis in HCC, and in vitro blockade of miR-135a significantly reduced the PVTT incidence, suggesting a potential application of miR-135a in PVTT therapy [93]. Moreover, miRNA-135a is highly expressed in HCC with recurrence within 12 months after resection. Therefore, the high intratumoral miRNA-135a expression could be a potential indicator for adjuvant therapy post-resection [92]. Likewise, deficiency in the serum level of vit D is reported with advanced stages of HCC and poor outcome [94,95,96,97,98]. Additionally, the serum level of IL-13 was higher in the HCC group and associated with an activated status of circulating monocytes [99,100,101]. 

Interestingly, high HCV load was a risk factor for therapy failure and the development of HCC in non-responders. Similarly, Noh et al. reported that serum HCV viral load is a risk factor for the development of HCC but not liver-related mortality [102]. Higher HCV RNA load could lead to a higher HCC prevalence [102]. DAA-induced SVR causes a significant reduction (about 71%) in HCC risk. Treatment with DAAs is not associated with increased HCC risk compared with treatment with IFN [103]. In addition, DAA therapy failure was recorded in HCV-infected patients with active HCC tumor at the start of therapy. However, SVR was achieved in those patients after administration of HCC curative therapies [104]. Other study showed that the risk of “de novo” HCC in advanced HCV-infected patients receiving DAA therapy, was not higher, and might be lower, than that of untreated patients during the first year. Moreover, the risk of HCC was further declined progressively with time after a SVR was achieved [105]. In contrast, other study suggests that the higher incidence of HCC following SVR with IFN-free therapy relates to baseline risk factors/patient selection, and not the use of IFN-free therapy [106]. Moreover, Calvaruso, et al. [107] found that the SVR to DAA treatment decreased the incidence of HCC over a mean follow-up of 14 months. Finally, SVR was associated with a considerable reduction in the risk of HCC. Kanwal et al. reported that DAAs did not promote HCC. However, in patients with SVR, the absolute risk of HCC remained high in patients with established cirrhosis [108].

ROC analyses for serum IL-13, miRNA-135a, and vit D revealed that IL-13 (>135 pg/mL) has better prognostic accuracy for predicting the treatment failure with 100% sensitivity and 96% specificity, followed by miRNA 135a with 97% sensitivity and 91% specificity. In addition, serum IL-13 (>249 pg/mL) can predict the development of HCC with 100% sensitivity and specificity of 98% and 89% for IL-13 and miRNA 135a, respectively. These markers can be diagnostically useful for the clinician to predict the therapy outcomes and the possibility of HCC development, especially in non-responders. Clinicians can benefit from the markers to take the appropriate preventive measures to reduce the complications associated with the treatment failure. It is important to highlight that the level of these markers could differ at other places depending on the patient criteria, cohort size, instruments, and methodology.

The manuscript has number of advantages including the recruitment of large number of participants who received HCV-DAA therapy, association between some selected parameters (IL13, vit D and micoRNA 135a) and HCV treatment outcome and development of HCC. On the other hand, this study also has some limitations. The exact mechanism of resistance to the DAA was not analyzed. We analyzed the changes in IL13 only; however, the modulation of the other pro and anti-inflammatory cytokines was not investigated. In addition, the association between response to therapy and other micro-RNA was not analyzed. Furthermore, these parameters were assessed in patients recruited from one center. Moreover, the enrolled patients received one approved regimen (sofosbuvir and daclatasvir ± ribavirin). Future studies including patients from multi-centers and who received different HCV DAA regimens are needed to ascertain these findings.

## 5. Conclusions

We reported the prognostic markers of IL-13, miRNA135a, and vit D associated with DAA treatment failure in chronic HCV-infected patients receiving sofosbuvir and daclatasvir ± ribavirin. Additionally, we showed that these markers could predict HCC development following failure in DAA therapy.

## Figures and Tables

**Table 1:** Sociodemographic and clinical characteristics of the study population.

	Responders (*n =* 880)	Non Responders (*n =* 70)	Controls (*n =* 50)	*p* Value Comparison 3 Groups	*p* Value Comparison Responders vs. Non Responders
Age	47.83 ± 9.78 (30–70)	50.14 ± 9.5 (32–71)	49.62 ± 9.024 (33–69)	0.082	0.0549
Sex M/F	418/462 (47.5/52.5)	33/37 (47.1/52.9)	30/20 (60/40)	0.224	0.99
Smoking	286 (32.5)	20 (28.6)	0	<0.001	0.51
Comorbidities	528 (60)	57 (81.4)	0	<0.001	<0.001
Severity of liver disease Non-cirrhotic Cirrhotic	748 (85) 132 (15)	26 (37.1) 44 (62.9)	0 0	<0.001	<0.001
HCC incidence	0	25 (35.7)	0	<0.001	<0.001
IL-13 (pg/mL)	102 ± 20.6 (24–187)	308.9 ± 113.4 (136–542)	75.2 ± 4.8 (58–81)	<0.001	<0.001
miRNA-135a (fold change)	2.1 ± 0.9 (0.4–4.6)	4.6 ± 0.7 (2.7–6.7)	1.2 ± 0.25 (0.7–1.6)	<0.001	<0.001
VIT D (ng/mL)	32.2 (2.5–71)	12.65 (2–45)	44.1 (30–70)	<0.001	<0.001
HCV-RNA (IU/mL)	4.7 × 10^5^ (0.8 × 10^3^–6 × 10^8^)	9.6 × 10^6^ (1.2 × 10^4^–1.26 × 10^8^)	-	-	<0.001

Values are presented as mean ± standard deviation (range), median (minimum-maximum) or n (%) unless otherwise indicated. *p*-value < 0.05 means significant. For comparison between the groups one-way ANOVA analysis or Kruskal–Wallis and Mann–Whitney or Student’s *t*-test is used to compare between the two groups. HCC: hepatocellular carcinoma. IL-13: Interleukin 13. Vit D: Vitamin D. Comorbidities: Hypertension, DM.

**Table 2:** Comparison between non-responders with and without HCC.

	Non Responders without HCC (*n =* 45)	Non Responders with HCC (*n =* 25)	*p* Value
Age (years)	49.71 ± 9.37 (32–71)	50.92 ± 9.798 (34–69)	0.49
Sex M/F	23/22 (51.1/48.9)	10/15(40/60)	0.45
Smoking	12 (26.7 %)	8 (32 %)	0.78
Comorbidities	34 (75.6 %)	23 (92 %)	0.116
IL-13 (pg/mL)	262.8 ± 106.3 (136–532)	391.8 ± 71.5 (267–542)	<0.001
miRNA-135a (fold change)	4.5 ± 0.8 (2.7–6.7)	4.8 ± 0.5 (3.7–5.67)	0.0426
VIT D (ng/mL)	14.7 (2–45)	11.97 (2–41)	0.13
HCV-RNA (IU/mL)	4.6 × 10^6^ (1.21 × 10^4^–4.2 × 10^7^)	1.8 × 10^7^ (3.7 × 10^6^–1.26 × 10^8^)	0.002

Values are presented as mean ± standard deviation (range), median (minimum − maximum) or n (%) unless otherwise indicated. *p*-value < 0.05 means significant. for comparison as determined by Mann–Whitney tests or Student’s *t*-test.

**Table 3:** Diagnostic accuracy of IL-13, miRNA-135a, and vit D to predict treatment failure and HCC with the best predictive cut offs.

	AUC (95%CI)	SE	SP	+LR
(a) For prediction of DAA treatment failure				
IL-13 (>135.9 pg/mL)	0.999 (0.998–1)	100	96.1	25.9
miRNA135a (>3.562-fold change)	0.978 (0.968–0.989)	97.14	91.59	11.7
Vit D (<21.13 ng/mL)	0.851 (0.807–0.895)	82.9	82.7	4.8
(b) For prediction of HCC development				
IL-13 (>249 pg/mL)	0.992 (0.987–0.997)	100	98.4	61.7
miRNA135a (>3.66 fold change)	0.976 (0.964–0.989)	100	88.96	9.06
Vit D (<21.04 ng/mL)	0.872 (0.809–0.935)	92	79.9	4.6
(c) For prediction of DAA treatment failure in non-cirrhotic patients				
IL-13 (>135.9 pg/mL)	0.998 (0.995–1.000)	100	96.9	32.5
mRNA135a (>3.562-fold change)	0.967 (0.950–0.983)	96.2	91.7	11.6
Vit D (<24.11 ng/mL)	0.861 (0.780–0.942)	84.6	83.7	5.2
(d) For prediction of DAA treatment failure in cirrhotic patients				
Il-13 (>166.3 pg/mL)	1 (0.979–1.000)	100	100	-
mRNA135a (>3.643 fold change)	0.985 (0.971–1.000)	97.73	92.42	12.9
Vit D (<22.5 ng/mL)	0.657 (0.573–0.742)	86.4	50	1.7
(e) For prediction of HCC development in cirrhotic patients				
Il-13 (>232.5 pg/mL)	0.949 (0.9184–0.9798)	100	90.1	10.1
mRNA135a (>3.643 fold change)	0.936 (0.900–0.972)	100	81.46	5.4
Vit D (<22.5 ng/mL)	0.6575 (0.558–0.757)	92	46.4	1.7

AUC: area under the curve; CI: confidence interval; +LR: positive likelihood ratio, SE: sensitivity; SP: specificity.

## Data Availability

All the data are present in the main text.
